# A retrospective epidemiological study of sarcoptic mange in koalas (*Phascolarctos cinereus*) using wildlife carer admission records

**DOI:** 10.1016/j.ijppaw.2024.100955

**Published:** 2024-06-13

**Authors:** Ellyssia T. Young, David Phalen, Aaron C. Greenville, Kylie Donkers, Scott Carver

**Affiliations:** a School of Life and Environmental Sciences, University of Sydney, Sydney, New South Wales, 2050, Australia; b Sydney School of Veterinary Science, Faculty of Science, The University of Sydney, Sydney, New South Wales, 2050, Australia; c Dutch Thunder Wildlife Shelter, Victoria, 3644, Australia; d Department of Biological Sciences, University of Tasmania, Hobart, Tasmania, 7005, Australia

**Keywords:** Koala, *Phascolarctos cinereus*, Sarcoptic mange, *Sarcoptes scabiei*, Wildlife carer

## Abstract

Outbreaks of sarcoptic mange are sporadically reported in koala populations across Australia, but disease characteristics (e.g., distribution across the body) remain poorly understood. In an area of Northern Victoria regular cases coming into care suggest mange may have become enzootic, and here we characterise those koala mange admission records. In 18% (n = 10) of mange affected koala reports that had a recorded outcome (n = 55), the animals died before the carers could locate them, and of the remaining 45 koalas that were alive upon carer arrival, 80% (n = 36) had to be euthanised due to severe mange. The number of admissions varied among years (highest observed in 2019), and over 60% of affected koala admissions were male. Male admissions peaked in austral spring and again in late austral summer-autumn (mating and birthing seasons), with female admissions only exhibiting the latter peak (birthing season). Fissures of the epidermis of the front paws occurred in 100% of admitted koalas, with 70% also showing these signs elsewhere on ventral surfaces or limbs. Only male koalas had signs of mange on the chest and face, and only female koalas had signs of mange on their back. Collectively, this study suggests sarcoptic mange can be a severe disease in koalas, and that male koalas may play an important role in seasonal transmission dynamics. We discuss how these findings may help inform intervention strategies.

## Highlights

• This study sought to investigate the epidemiology of sarcoptic mange disease in a population of koalas in Northern Victoria retrospectively using detailed carer admission records.
• Out of 45 koala admissions with sarcoptic mange that were alive when carers arrived, 80% (n = 36) of these had to be euthanised due to advanced mange.
• Over 60% of koala mange admissions were male each year, and male admissions peaked in austral spring and again in late austral summer-autumn, with female admissions only exhibiting the latter peak.
• There were different patterns of sarcoptic mange signs between male and female koala.
• Collectively, this study suggests sarcoptic mange can be severe in koala, and that male koala may play an important role in seasonal transmission dynamics. Our findings may help to inform disease management plans.

## Graphical abstract

**Figure:**
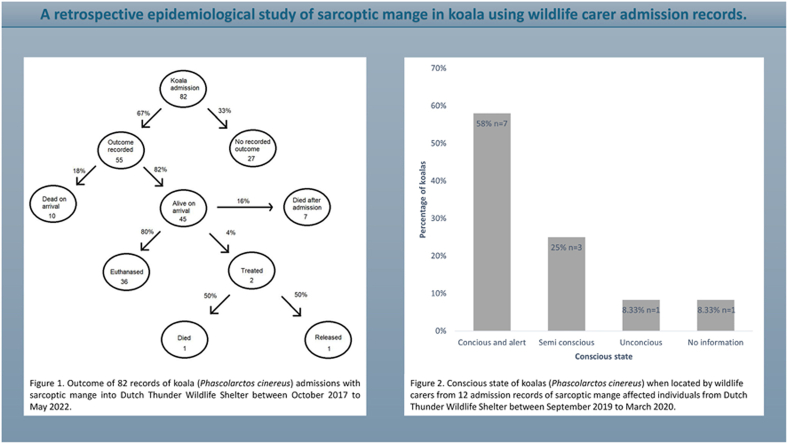

## 1 Introduction

Sarcoptic mange is a highly contagious skin disease, caused by the *Sarcoptes scabiei* mite, and has been documented to infect a wide range of mammalian host species worldwide (Escobar et al., 2022, Pence and Ueckermann, 2002). Within Australia, *S. scabiei* has become geographically widespread, but historically was only reported in a limited number of mammal species (Fraser et al., 2016). Thought to have been introduced to Australia by humans and their domestic animals, sarcoptic mange is now one of the most significant parasitic diseases of Australian marsupials (Martin et al., 2017). Sarcoptic mange is an enzootic disease in many bare-nosed wombat (*Vombatus ursinus*) populations across Australia (Martin et al., 2017), and occasional outbreaks have been reported in southern hairy nosed wombats (*Lasiorhinus latifrons*), wallabies (*Wallabia bicolor* and *Notamacropus agilis*) (McLelland and Youl 2005; Holz et al., 2011), southern-brown bandicoots and quenda (*Isoodon obesulus* and *Isoodon fusciventer*) (Wicks et al., 2007; Botten et al., 2022), and koalas (*Phascolarctos cinereus*) (Speight et al., 2017). Furthermore, there is evidence to suggest that sarcoptic mange may be becoming endemic in some of these species as well, such as koalas (Speight et al., 2017), and quenda (Botten et al., 2022).

The first recorded case of sarcoptic mange in koalas occurred in Victoria in 1974 and consisted of one hand-reared juvenile koala which had been raised with a bare-nosed wombat, so it was believed to have acquired sarcoptic mange from the wombat (Barker 1974). In 1980, there was an outbreak of sarcoptic mange in a captive koala population in Queensland which was linked with the arrival of a wild koala (Brown et al., 1982). In 1983 a study of diseases in koalas found two out of 55 deceased koalas submitted for necropsy were affected by sarcoptic mange (3.6%) (Obendorf 1983). Sarcoptic mange has been recently reported in a number of geographically dispersed populations across Victoria and South Australia, with the significance for conservation not well understood (Speight et al. 2017, 2018).

Sarcoptic mange in koalas presents as crusted mange which is characterised as intense pruritis leading to hyperkeratotic lesions particularly on the face, chin, stomach, limbs and paws of affected individuals, which can result in thickening of the skin, fissuring, emaciation and death possibly owing to secondary infections (Speight et al., 2017). Sarcoptic mange can lead to significant welfare concerns, however it is a treatable disease, with currently the most effective long-lasting treatment in wombats being fluralaner (Wilkinson et al., 2021) and this may also be therapeutic in infected koalas (Watts et al. in prep). Very little is known about the epidemiological characteristics of mange outbreaks in koalas (Speight et al., 2017). For effective management of a disease, it is imperative to understand the characteristics of the infection.

Koalas are not currently listed as of conservation concern in Victoria, but are listed as Endangered in NSW, QLD and the ACT under the Environment Protection and Biodiversity Conservation Act (1999) (Department of the Environment 2022), due to the species vulnerability to environmental and anthropogenic pressures (McAlpine et al., 2015). Along with the ecological value of this species, the visible effects of the disease on an iconic species such as the koalas means there is social demand to intervene and find solutions (Carver 2016). In this study we utilize wildlife carer records to retrospectively investigate sarcoptic mange in free-ranging koalas from Northern Victoria, describing the characteristics and seasonal patterns of sarcoptic mange. We aim to contribute to the understanding of sarcoptic mange in koalas and advance understanding on areas where disease management could potentially be improved.

## 2 Material and methods

### 2.1 Study area

Koala admission records used for this study came from Dutch Thunder Wildlife Shelter in Northern Victoria, which acts with a license issued under the Wildlife Act 1975 (Wildlife Shelter license number 14321755). This license permits the wildlife carer to undertake activities such as possess, pursue, capture, and/or euthanise and release protected wildlife in compliance with the standards outlined in the Act. Because this study consisted of analysis of existing data, it did not require Animal Ethics approval.

**Fig. 1:**
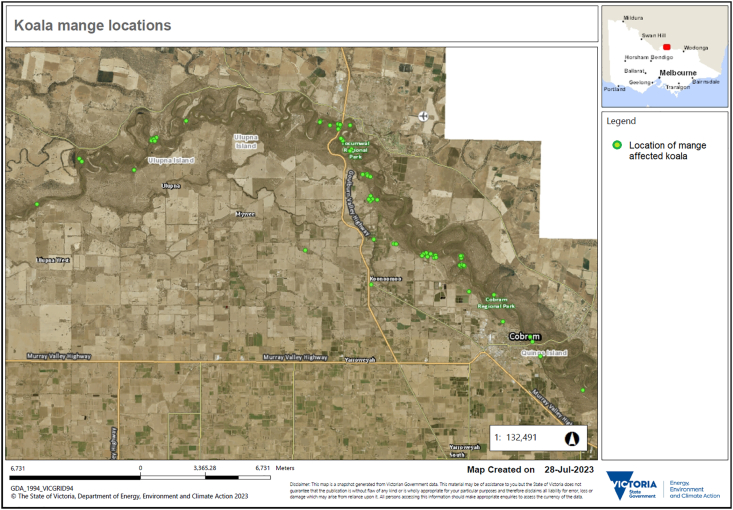
Map of locations of koalas (Phascolarctos cinereus) admitted with sarcoptic mange.

Records were available from October 2017 to May 2022. Admission data included variables: date, location found, weight, sex, clinical presentation, and outcome. Most koalas were found in remnant riverine woodland (Fig. 1). All sarcoptic mange diagnosis were made by the experienced wildlife carer with vet nurse training using clinical signs and where there was uncertainty, microscopic observation of skin scrapings were used to confirm diagnosis. Age was assigned based on visual observation of the whole animal, including body size and overall condition. Koalas were classified as either dependent joey, sub-adult, adult or geriatric, and if the animal was euthanised tooth wear was used to confirm age. However, for the purposes of this study koala age was simplified as dependent joey or adult (sub-adult, adult and geriatric). There was some variation in the extent of information among admission records and consequently sample sizes for descriptive analysis. For a subset of the records (n = 12/82) a detailed assessment of the distribution and severity of mange over a koala's body could be made. To assess distribution and severity of mange the koala's body was divided into 13 segments and scored for presence or absence of mange signs (e.g., evidence of pruritis, alopecia, hyperkeratosis, and skin fissuring). Other clinical signs of ill health, such as diarrhea and emaciation were not included in severity scoring or distribution of mange assessment. A percentage of body affected was calculated based on how many segments of the body had mange signs present (e.g., 2/13 segments affected equates to 15%of body affected by mange). More detailed severity scoring was not possible from the data available. Body segments were chest, stomach, testicles or pouch, upper hind legs, lower hind legs, hind paw, chin, front paw, lower front leg, upper front leg, ears, head and back. This scoring was then used to determine the percentage of the body affected by sarcoptic mange and indicate the severity of the sarcoptic mange signs in each animal. To evaluate koala mange admission patterns through time, we undertook a descriptive analysis of the data among calendar months, austral seasons and surrounding the koala breeding seasons (mating season August to November, birthing season December to March, non-breeding season April to July (Smith 1980a; McLean and Handasyde 2006)).

## 3 Results

### 3.1 Characteristics of koala mange admissions

**Fig. 2:**
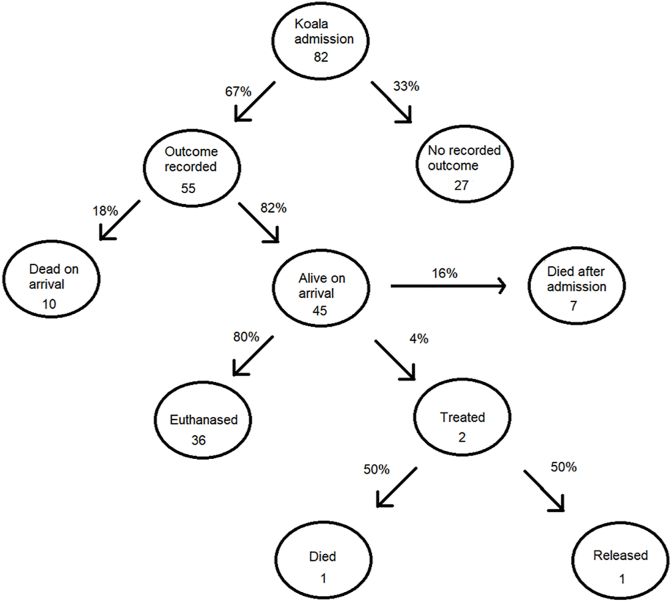
Outcome of 82 records of koala admissions with sarcoptic mange into Dutch Thunder Wildlife Shelter between October 2017 to May 2022.

We undertook a descriptive analysis of koala admission records and describe patterns in the numbers and characteristics of koalas admitted with sarcoptic mange. The records consisted of 82 koala admissions with sarcoptic mange and occurred between October 2017 and May 2022 (Fig. 2). Of the total 82 admission records 55 (67%, n = 82) had a recorded outcome. The admission records with no outcome (n = 27) were only excluded from analysis of outcome. Of these 55 with recorded outcomes, 10 (18%, n = 55) koalas died prior to the carer's arrival, and 45 (82%, n = 55) were alive upon the carer's arrival (Fig. 2). Out of the total koala mange admissions (n = 82), 45 of the koalas that were alive on the carer's arrival, of which 36 (80%, n = 45) were euthanised on site by an experienced wildlife carer due to advanced stages of disease, 7 (16%, n = 45) died after admission to the rehabilitation centre and 2 (4%, n = 45) koalas were treated for sarcoptic mange. Of the 2 koalas that were treated, both received supportive therapies upon admission as deemed appropriate by the wildlife carer, one male koala recovered following treatment with Cydectin® pour on (Virbac Australia) (5 g/L moxidectin), 3 mls per treatment weekly for 4 weeks, and was successfully released. The second male koala was a severe case of sarcoptic mange with a questionable chance of recovery. After much consultation with a wildlife veterinary specialist, treatment with a single dose of Bravecto® Spot-on (MSD Animal Health) (136.4 g/kg fluralaner) was attempted on the severely diseased koala, but the individual died overnight. No post-mortem was completed due to the obvious advanced extent of disease, and it was assumed to have died of mange associated secondary infections.

**Fig. 3:**
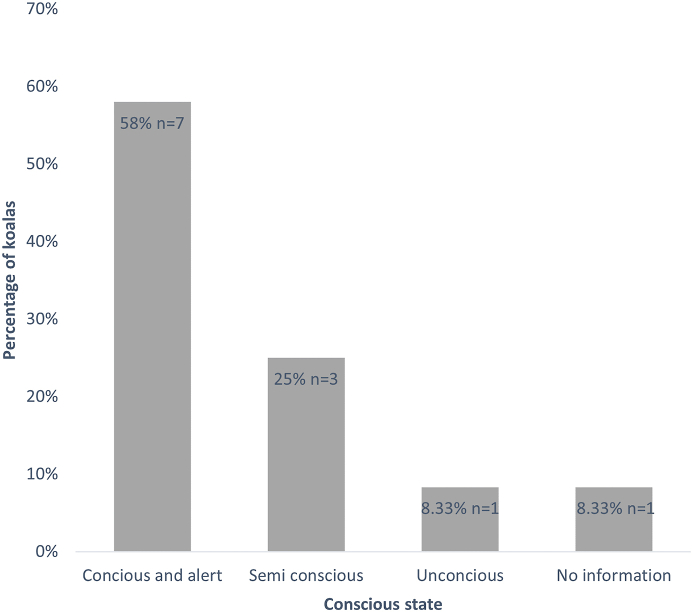
Conscious state of koalas when located by wildlife carers from 12 admission records of sarcoptic mange affected individuals from Dutch Thunder Wildlife Shelter between September 2019 to March 2020. Percentages and sample sizes are provided for each category.

In 22% (n = 12) of the 55 records which were alive on arrival, detailed notes were made of the state of koalas upon encounter (Fig. 3). Of these 12 koalas, 7 (58.3%) were conscious and alert upon collection by the wildlife rehabilitator, 3 (25%) were semi-conscious, and 1 (8.3%) were unconscious and 1 (8.3%) had no conscious state recorded (Fig. 3). Unfortunately, information on evidence of co-infections was extremely limited. One koala was recorded to have urine stains but was not tested further, another had injuries noted and one koala was recorded to have no other health issues. The remaining 79 cases did not have any comments on co-infections or other health issues.

### 3.2 Demographics and annual patterns of koala admissions

There were proportionally more male (66%, n = 82) then female (28%, n = 82) koalas admitted with signs of sarcoptic mange, with 6% of records having no sex listed (n = 82) (Table 1). Of the 23 females admitted, 3 had pouch young (13%, n = 23). Of these 3 pouch young, 1 was recorded to have sarcoptic mange, and 2 had no record of sarcoptic mange. The mother of the pouch young that was recorded was also recorded to have mange. We analysed the temporal patterns of koalas coming into care with sarcoptic mange. Analysis was restricted to 2018–2021 (n = 75), as records for years 2017 and 2022 were incomplete. The highest number of cases of koalas coming into care suffering sarcoptic mange were documented in 2018 and 2019, with 2019 having the highest number of records (n = 27) (Table 1). Each year there were more males than females reported with sarcoptic mange, the largest difference was in 2018 where 83% (15 males out of 18 admissions) of the recorded sarcoptic mange affected koalas were male. Male koalas accounted for over 60% of sarcoptic mange records each year.

**Table 1:** Breakdown of admission records of koalas (Phascolarctos cinereus) affected by sarcoptic mange from 2018 to 2021. Admission record data from 2017 to 2022 were incomplete and excluded from individual analysis.

	2018	2019	2020	2021	Average across years
Male	83%	63%	69%	64%	**70 %**
Female	6%	37%	25%	29%	**24%**
No record	11%	0%	6%	7%	**6%**
Total number of admissions	18	27	16	14	**82**

### 3.3 Monthly, seasonal and breeding cycle patterns in mange

**Fig. 4:**
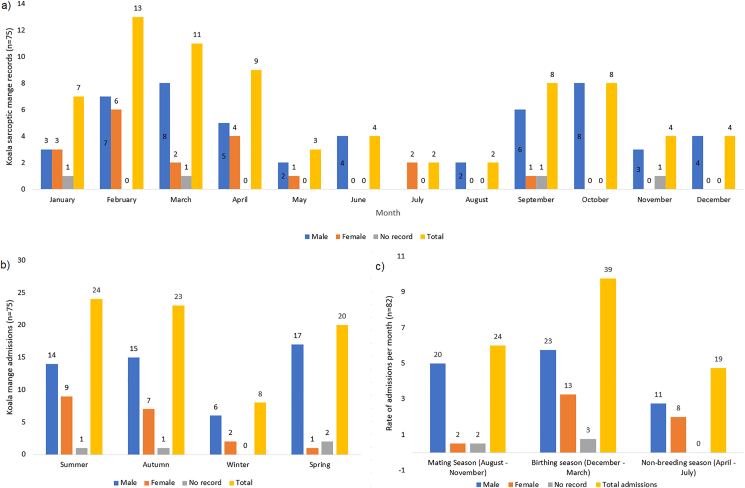
Koala sarcoptic mange admissions: a) Number of cases of koalas per month from 75 koala admission records affected by sarcoptic mange from January 2018 and December 2021. b) Number of koala sarcoptic mange admissions per season (n = 75). c) Rate of koala sarcoptic mange admissions per month for each koala breeding season (n = 82).

The highest numbers of koala sarcoptic mange records were recorded in late Austral summer and early autumn (February–April) (n = 75). Two distinct peaks in koala sarcoptic mange admissions were evident, from February–April and September–October (Fig. 4a). Mange prevalence increased in males in early spring, beginning in September and peaking in October and again in February/March (Fig. 4a and b). In contrast the early spring peak in sarcoptic mange prevalence was not observed in females, increased admissions were observed in January and peaked in February, and cases did not reduce until May (Fig. 4a and b). There were no records of sarcoptic mange that affected female admissions across all years in June, August, October or December, whereas the only month with no admissions of males with sarcoptic mange was July (Fig. 4a). We then analysed the rate of koala mange admissions (n = 82) per month in each breeding season (mating, birthing, non-breeding) (admissions divided by the number of months in each breeding season), with the highest rate of total admissions per month occurring in the birthing season (December to March) (9.75, n = 39), followed by the mating season (August to November) (6, n = 24) and then the non-breeding season (April to July) (4.75, n = 19) (Fig. 4c). When separated by sex, the highest rate of both male and female koala admissions occurred in the birthing season (5.75, n = 23 and 3.25, n = 13 respectively). However, within each season (mating, birthing and non-breeding) admission rate was highest for males (5, n = 20; 5.75, n = 23 and 2.75, n = 11 per season respectively).

### 3.4 Sarcoptic mange presentation and severity

**Fig. 5:**
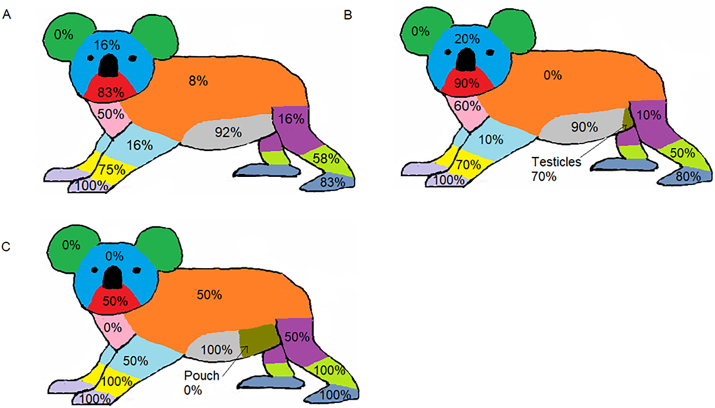
Frequency of sarcoptic mange infestation by body region recorded from 12 koalas that were reported with mange between September 2019 to March 2020. **A)** Total records. Sex specific features (pouch, testicles) were included as the stomach region. **B)** Male records (n = 10), **C)** Female records (n = 2).

Of the 55 admission records which were alive on arrival, 12 records contained more detailed information which allowed further interrogation and were used to investigate sarcoptic mange presentation and severity (subset data). Skin fissuring was the most recorded sign of sarcoptic mange with 42% (n = 12) of admissions showing these signs (Table 2). Another 8% (n = 12) of admissions listed clinical signs as present but no specific symptom was given (Table 2). Emaciation and skin crusting were both recorded in 25% of admissions (n = 12), with pruritis, alopecia and diarrhea recorded in only 8% (n = 12) of cases. Of the 12 admissions that recorded part of body affected, the most frequently affected body region observed was the front paw with 100% (n = 12) of koalas reported to show signs of sarcoptic mange on this body region. Stomach, hind paw, chin and lower fore limb were recorded to be affected by sarcoptic mange in over 70% of cases (n = 12). No records noted sarcoptic mange on the pouch (females only, n = 2), ears or head (both males and females, n = 12). When analysed by sex, only males (n = 10) were observed to have sarcoptic mange on their chest and head segments and only females (n = 2) were observed to have sarcoptic mange affecting their back (Fig. 5). Both females were recorded to have had sarcoptic mange on their stomach, upper hind legs, hind paw, front paws, and lower fore limb (100%, n = 2). Whereas all males (n = 10) were only reported to have sarcoptic mange on their front paws.

**Table 2:** Frequency of clinical signs recorded in 12 koalas rescued with sarcoptic mange in Northern Victoria between September 2019 and March 2020.

Clinical signs	Frequency of detection (%)
Fissuring	42%
Emaciation	25%
Hyperkeratotic lesions/Skin crusting	25%
Pruritis	8%
Alopecia	8%
Diarrhea	8%
Clinical signs noted but no specific sign recorded	50%

## 4 Discussion

The epidemiology of sarcoptic mange disease can be highly variable among host species and even among populations of the same species (Browne et al., 2022). Patterns of disease may range from epizootic to enzootic (Escobar et al., 2022). The occurrence of sarcoptic mange disease in the koalas of the study area showed strong seasonal patterns. The highest numbers of koala sarcoptic mange cases were recorded in late Austral summer and early autumn (February–April). High prevalence of sarcoptic mange in males was observed in September/October (in the mating season Smith 1980a) and then again in February/March (the birthing season McLean and Handasyde 2006), whereas in females’ high prevalence was only observed in January/February (the birthing season McLean and Handasyde 2006) with cases reducing by May (non-breeding season McLean and Handasyde 2006). The increase in male-male interactions during territorial disputes and competition as well as increased exposure to potentially contaminated fomites in the mating season is a likely explanation for the increase in sarcoptic mange admissions in males due to increased risk of mange transmission between individuals and the environment (Speight et al., 2017). The increased activity demonstrated by males during late winter and early spring (Watchorn and Whisson 2019) and scent marking behaviour (which occurs throughout the year), where males rub the scent gland on their chest on trees to mark territory and communicate with other koalas (Smith 1980b; Mitchell 1990), may also increase their risk of acquiring sarcoptic mange mites from environmental fomites or other host species. The relative importance of fomites and specific sources of environmental reservoirs of mange mites has not yet been comprehensively investigated for koalas, but bark around the base of trees has previously been proposed as a possible exposure source to *S. scabiei*, owing to the potential of other mangy mammals (e.g. fox or wombat) to rub against these and deposit mites (Speight et al., 2017). Occurrence of mange affected females increased later in the year (the birthing season). Sarcoptic mange progression in koalas is not currently well understood, particularly relative to some other important disease issues this species faces (Robbins et al., 2020). Based on what is known about mange progression in the closely related wombat (Skerratt 2003), it is feasible that female koalas exposed to sarcoptic mites during the mating season (from August to November) may develop visual signs of sarcoptic mange by January/February. The high rate of female koala admissions in the birthing season and the low numbers of dependent joeys (3 out of 13 females had joeys recorded) warrants further research.

Alopecia is a commonly occurring symptom of sarcoptic mange in many affected species with varying degrees of alopecia observed between hosts, and generally quantified as an observable proportion of hair loss (Martin et al., 2018; Van Wick and Hashem 2019; Montecino-Latorre et al., 2020; Botten et al., 2022). The koalas in this study displayed low levels of alopecia in comparison to many other impacted species, either closely or distantly related (Martin et al., 2018; Lewin et al., 2023). In this study, fissuring, skin crusting, and emaciation were more commonly observed sign of mange in koalas. This is not unique to koalas, for example Iberian lynx exhibit limited alopecia associated with sarcoptic mange (Oleaga et al., 2019). This lack of a highly visual clinical sign can have implications for detection and management of mange within free-ranging koalas. More research is needed to understand how best to assess mange among free-ranging koalas, and if the persistence and severity of sarcoptic mange are associated with anthropogenic land use, host specific physiological characteristics, comorbidities or immunological naivety.

Consistently there were higher numbers of male koalas admitted with sarcoptic mange across all years, suggesting that male koalas are more likely to be infested with sarcoptic mange. A higher proportion of male koalas affected by mange has also been observed in a previous study (Speight et al., 2018). Early in the breeding season male koalas are more active looking for females and often engage in territorial behaviour which may involve close contact (Jiang et al., 2022). Due to increased activity early in the breeding season (austral spring) (Ryan et al., 2013), male koalas may have greater susceptibility and exposure to mange via direct transmission or to environmental fomites harbouring mange mites through activities such as scent marking. This along with the effect of anthropogenic and environmental stressors that koalas face (Narayan 2019) may reduce immune defence mechanisms against parasitic disease. The presence of other diseases may increase the animal's risk of developing mange once exposed to the *S. scabiei* mites for example koala retrovirus, however specific comorbidities of mange infection is still unknown (Tarlinton et al., 2005; Greenwood et al., 2023). It would be valuable to have a feasible mechanism that would support wildlife carers to collect and submit samples that could be used for further analyses. The increase in male interactions at the start of the breeding season in spring could be an important driver in sarcoptic mange dynamics and persistence in this population and warrants further investigation.

Interestingly, there were sex-specific patterns of clinical signs observed within the koala admissions with sarcoptic mange. These findings are inherently tentative, owing to the small number of females for which this information was available. All males were reported to have signs on their chest and face. Since mange signs spread from the site of infection as the disease progresses (Skerratt 2003), this supports the idea that interactions between males during the mating season when males may be exhibiting scent marking behaviour and increased male-male interactions, is likely a key method of transmission. Both females were recorded to have sarcoptic mange signs on their backs, and disease progression characteristics as well as the increase in female sarcoptic mange cases in summer suggests that mating, may be a key form of transmission in female koalas. Other studies have documented sexual differences in the prevalence and visible effects of sarcoptic mange infection (Pence and Windberg 1994; López-Olvera et al., 2015; Speight et al., 2017), so is not unique to koala, and may help to understand transmission dynamics in this species. Currently it is believed that another important mode of transmission of sarcoptic mange among koalas is the persistence of *S. scabiei* mites from environmental fomites (Speight et al., 2017), and this is supported by this study as 100% of the koala admissions exhibited mange signs on their front paws, however sources and dynamics of environmental contamination requires further research in koalas. One case where a female with a joey both presented with mange signs suggests that vertical transmission between mothers and offspring also plays a role in transmission dynamics, but the relative importance cannot be fully understood from this study due to scarcity of applicable admission records. While conclusions from this study are tentative due to the small number of females represented, the patterns of sarcoptic mange signs observed suggest that transmission is complex and multi-factorial, and behaviours associated with mating including male-male aggression and mating itself may be important methods of transmission of infection in this population.

The highly visual nature of alopecia is commonly the observation used to identify sarcoptic mange affected animals for disease management programs and welfare assessments (Simpson et al., 2016; Montecino-Latorre et al., 2020; Sannö et al., 2021). The form of disease presentation observed in koalas with characteristic low levels of alopecia makes spotting sick individuals difficult. Indeed, the reclusive and arboreal nature of koalas makes spotting and assessing them for disease intrinsically harder. Consequently, by the time affected koalas are identified and admitted into care they may be at an advanced stage of disease, so treatment success may be more challenging. A recent study shows how koalas can have variable and often elevated stress levels during rehabilitation (Charalambous et al., 2021) and this along with more advanced stages of sarcoptic mange and the associated physical effects of this disease may hinder a koala's ability to recover following treatment. Specific population level factors such as genetic diversity and inbreeding may predispose koalas to have higher stress responses as well as disease susceptibility (Phillips 2018). This, along with potential disease induced stress (Pérez et al., 2019) may further complicate decisions as to whether it is ethical to bring koalas into care to undergo treatment, or alternatively euthanise the animal and prevent further welfare compromise.

It is possible that the COVID pandemic may have influenced koala admissions throughout the years of this study. However, it is unclear if this would have increased or decreased admissions and is deemed outside the scope of this study.

## 5 Conclusions

This study sought to investigate the epidemiology of sarcoptic mange disease in a population of koalas in Northern Victoria retrospectively using detailed carer admission records. We observed strong seasonal patterns in the prevalence of sarcoptic mange affected koalas admitted into care. Males showed two distinct peaks in mange prevalence, in the mating season (September/October) and then again later in the birthing season (February/March), whereas cases of females with sarcoptic mange only peaked in the birthing season (January/February). There were different patterns of sarcoptic mange signs between male and female koalas. The seasonal patterns and sex-specific distributions of sarcoptic mange suggest that males may be important drivers of sarcoptic mange dynamics within this population and that sarcoptic mange may have become enzootic in this population of koalas. Our findings demonstrate how severe mange can be in koalas and may help to inform disease management plans. This study was limited in sample size due to access to suitable admission records provided by wildlife carers and requires further investigation in other koala populations to gain a more complete understanding of koala sarcoptic mange epidemiology and immunology. There is a need to advance our understanding of what severity of mange can be effectively treated and requires an active survey of koalas to detect sick animals at earlier stages of disease. The amount of time for different mange severities to develop within an individual and within the population is currently unknown in this species so future directions should aim to further our understanding of clinical progression of mange signs and also the fine-scale landscape epidemiology of mange in koala.

## Funding source

Australian Postgraduate Award to EW.

## CRediT authorship contribution statement

**Ellyssia T. Young:** Writing – review & editing, Writing – original draft, Visualization, Resources, Project administration, Methodology, Investigation, Formal analysis, Data curation, Conceptualization. **David Phalen:** Writing – review & editing, Supervision, Conceptualization. **Aaron C. Greenville:** Writing – review & editing, Supervision. **Kylie Donkers:** Data curation. **Scott Carver:** Writing – review & editing, Supervision, Conceptualization.

## Declaration of competing interest

The authors declare that they have no known competing financial interests or personal relationships that could have appeared to influence the work reported in this paper.
